# *Lupinus albus* Protein Components Inhibit MMP-2 and MMP-9 Gelatinolytic Activity In Vitro and In Vivo

**DOI:** 10.3390/ijms222413286

**Published:** 2021-12-10

**Authors:** Joana Mota, Rosa Direito, João Rocha, João Fernandes, Bruno Sepodes, Maria Eduardo Figueira, Anabela Raymundo, Ana Lima, Ricardo Boavida Ferreira

**Affiliations:** 1 Linking Landscape, Environment, Agriculture and Food (LEAF) Research Center, Instituto Superior de Agronomia, University of Lisbon, 1649-004 Lisboa, Portugal; joana.mota.p@gmail.com (J.M.); jcfernandes@isa.utl.pt (J.F.); anabraymundo@isa.ulisboa.pt (A.R.); rbferreira@isa.utl.pt (R.B.F.); 2 Faculty of Veterinary Medicine, Lusófona University, 1749-024 Lisbon, Portugal; 3 Research Institute for Medicines and Pharmaceutical Sciences (iMed.UL), Faculty of Pharmacy, University of Lisbon, 1649-004 Lisbon, Portugal; rosadireito2008@gmail.com (R.D.); jrocha@ff.ulisboa.pt (J.R.); bsepodes@ff.ulisboa.pt (B.S.); efigueira@ff.ulisboa.pt (M.E.F.)

**Keywords:** *Lupinus albus* protein, MMP-9, MMP-2, gelatinases, MMP inhibitor, nutraceutical, gastrointestinal diseases

## Abstract

Matrix metalloproteinases 2 and 9 (MMP-2 and MMP-9) are regarded as important clinical targets due to their nodal-point role in inflammatory and oncological diseases. Here, we aimed at isolating and characterizing am MMP-2 and-9 inhibitor (MMPI) from *Lupinus albus* and at assessing its efficacy in vitro and in vivo. The protein was isolated using chromatographic and 2-D electrophoretic procedures and sequenced by using MALDI-TOF TOF and MS/MS analysis. In vitro MMP-2 and 9 inhibitions were determined on colon adenocarcinoma (HT29) cells, as well as by measuring the expression levels of genes related to these enzymes. Inhibitory activities were also confirmed in vivo using a model of experimental TNBS-induced colitis in mice, with oral administrations of 15 mg·kg^−1^. After chromatographic and electrophoretic isolation, the *L. albus* MMP-9 inhibitor was found to comprise a large fragment from δ-conglutin and, to a lower extent, small fragments of β-conglutin. In vitro studies showed that the MMPI successfully inhibited MMP-9 activity in a dose-dependent manner in colon cancer cells, with an IC50 of 10 µg·mL^−1^ without impairing gene expression nor cell growth. In vivo studies showed that the MMPI maintained its bioactivities when administered orally and significantly reduced colitis symptoms, along with a very significant inhibition of MMP-2 and -9 activities. Overall, results reveal a novel type of MMPI in lupine that is edible, proteinaceous in nature and soluble in water, and effective in vivo, suggesting a high potential application as a nutraceutical or a functional food in pathologies related to abnormally high MMP-9 activity in the digestive system.

## 1. Introduction

With today’s rise in chronic diseases, functional foods containing bioactive compounds that exhibit health-promoting effects are receiving increasing attention and have become a major trend for both consumers and the food industry [1]. This is particularly true for gastrointestinal diseases, such as cancer and inflammatory bowel diseases (IBDs), which are among some of the most diet-linked pathologies [2,3]. Under this context, the activity of molecules such as matrix metalloproteinases (MMPs), particularly gelatinases MMP-9 and MMP-2, has been recognized as major key players in inflammation [3,4] and in oncologic processes such as tumorigenesis, cell adhesion, and metastasis [5,6,7]. A considerably large body of evidence from pre-clinical and clinical tests shows that MMP-2 and MMP-9 inhibition can reduce both colorectal cancer (CRC) [3,8] and IBDs such as ulcerative colitis [5,8,9,10]. These findings have turned gelatinase inhibitors into very desirable pharmacological targets; however, previous efforts to target gelatinases MMP-2 and MMP-9 using broad-selective spectrum or semi-selective inhibitors (MMPIs) were hampered by dose-limiting toxicity, insufficient clinical benefits, and severe side effects due to their lack of specificity and inhibition of MMP-dependent physiological processes [11,12,13]. One method to overcome these constraints would be to discover non-toxic, more specific MMPIs capable of acting directly in loco, without the side effects reported in previous inhibitors [12,13]. Although this has yet to be discovered, a substantial amount of research has turned towards the discovery of plant-food derived MMPIs that may hold such features. Legume seeds are a good example, as they contain high levels of polyphenols, saponins, and protease inhibitors that have been reported to exhibit anticancer activities [14]. In fact, the considerably high number of reports on plant and food-based MMPIs concerns mostly secondary metabolites, such as flavonoids, alkaloids, and phenolic compounds [15]. However, these compounds present some constraints, since most of them can be destroyed during the digestive process and others are considered to be anti-nutrients, even exerting cytotoxicity at high levels. In our previous study, we analyzed and compared aqueous protein extracts from the seeds of eight different legume species that are usually consumed in Mediterranean diets and demonstrated that they inhibit MMPs and cancer cell migration [16]. Particularly, the water-soluble fraction of *Lupinus albus* seeds exhibited strong inhibitory activity on MMP-2 and MMP-9 in HT29 cells [17]. Reverse zymography revealed that this activity could be related to small polypeptides/proteins that seemed to be larger than other low molecular mass MMPIs also found in legume seeds, such as the Bowman–Birk or Kunitz inhibitors [18]. Recently, an up-scalable sequential method for producing a low molecular weight fraction for MMPI activity, using denaturing and precipitation steps, was developed [19] and patented (PCT International Patent Application No. PCT/EP2017/075020), demonstrating that MMPI can withstand heat and acid denaturation and suggesting a high potential as a nutraceutical in preventive diets. However, the nature and mode of action of these MMPIs remained to be characterized, particularly with respect to its activity validated in vivo after digestion.

Furthermore, although there is much evidence suggesting that several compounds from legume seeds can prevent cancer [20], with anticancer and anti-metastatic activities demonstrated in various animal models [14], only a few of them have been effectively isolated for nutraceutical purposes, and those that have, such as lunasin from soybean, exhibited no direct effect on MMPs [21]. Hence, compared to the latter, these new polypeptides from *L. albus* could be among the very few effective protein MMPIs found in legume seeds and may display various advantages as well as ease in isolation in larger amounts. Therefore, acknowledging that isolation and characterization of these polypeptides/proteins may open novel possibilities in the field of inflammatory diseases and cancer prevention, the goal of the present study is to identify and characterize these MMPIs from *L. albus* seeds and to evaluate their activities towards MMP-2 and MMP-9 in vitro and in vivo.

## 2. Results and Discussion

### 2.1. Isolation of the L. albus MMPI

**Figure 1:**
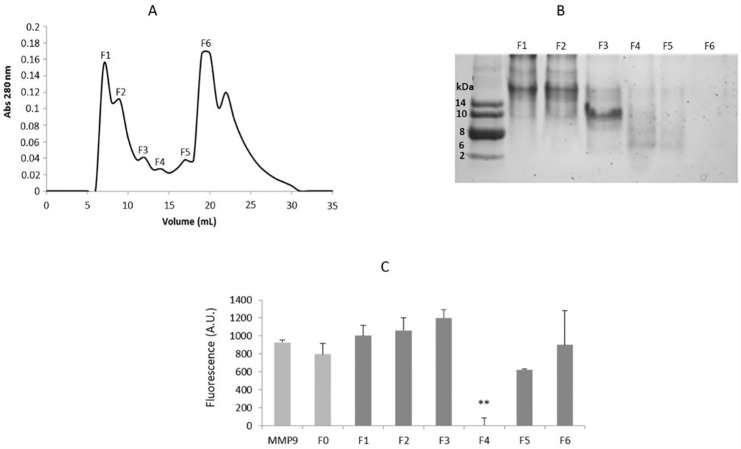
*L. albus* protein profile and corresponding MMP-9 inhibitory activity. (**A**) Size exclusion chromatography (SEC) in a Superdex 75 column of *L. albus* cotyledon total protein extracts. (**B**) Protein peaks were collected as fractions 1 to 6 and analyzed for polypeptide composition by SDS-PAGE. (**C**) MMP-9 proteolytic activity of fractions 1 to 6 obtained by SEC, as quantified by the DQ-gelatin method. Results are expressed in arbitrary units of fluorescence and represent an average of three replicates ± SD. ** *p* < 0.001 when compared to control.

In order to isolate the protein fractions found to be responsible for MMP-9 inhibition, the soluble protein pool of *L. albus* was fractionated by size-exclusion chromatography (SEC) and the individual fractions subsequently tested for inhibitory activity upon commercial MMP-9. Figure 1 shows the SEC protein profile obtained for the *L. albus* total soluble protein, with the corresponding polypeptide profiles of the collected fractions (1 to 6) analyzed by electrophoresis and their respective MMP-9 inhibitory activities as assessed by the Dye-quenched (DQ)-gelatin assay.

As observed in Figure 1, throughout the protein profile shown in Figure 1A, only fraction F4 presented significant MMP-9 inhibition (*p* < 0.001) and corresponded to a low molecular mass fraction (<20 kDa), which is in accordance with the study previously presented by Lima et al. [17], where reverse zymography results suggested that the MMPI protein/polypeptide had a molecular mass lower than 20 kDa.

### 2.2. Nature and Composition of the L. albus MMPI

**Figure 2:**
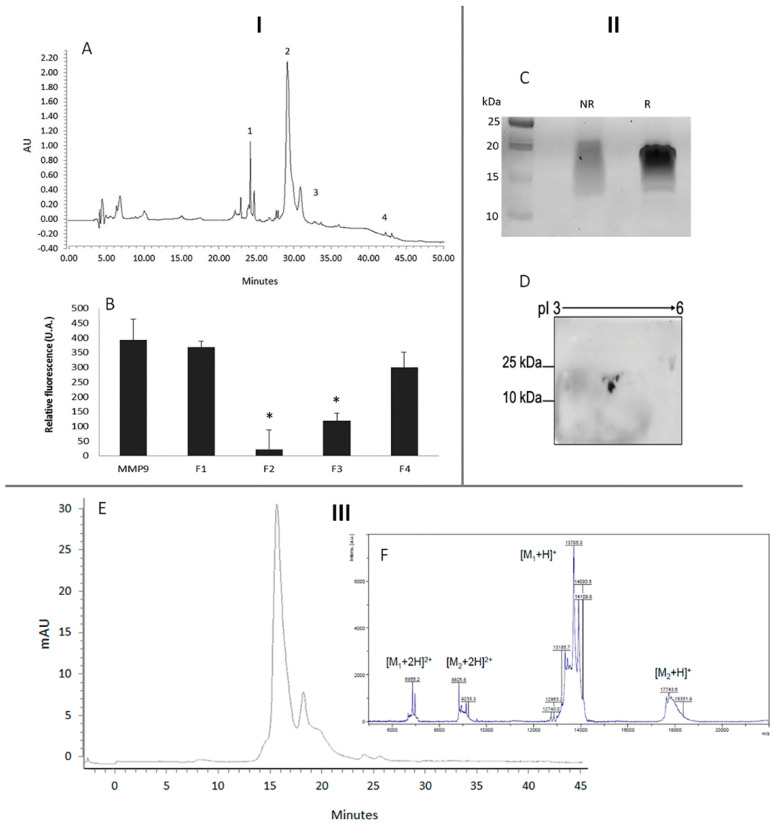
Characterization of the *L. albus* MMP-9 inhibitory protein. I: HPLC separation and peak purification of fraction 4 obtained by SEC (Figure 1A): (**A**) Reverse phase HPLC chromatographic profile of the MMP-9 inhibitory protein fraction 4 previously isolated from *L. albus* cotyledons by gel filtration. The main peaks obtained were collected as fractions 1 to 4. (**B**) MMP-9 proteolytic activity of fractions 1 to 4 in A, as quantified by the DQ-gelatin method. The results are expressed in arbitrary units of fluorescence and represent an average of three replicates ± SD. * *p* < 0.05. II: Electrophoretic analyses: (**C**) The polypeptide profile of fraction 2 in A was analyzed by denaturing electrophoresis performed under non-reducing (NR) and reducing (R) conditions. Representative image of the polypeptide composition of isolated MMPI from *L. albus* cotyledons separated by SDS-PAGE. (**D**) Two-dimensional electrophoretic separation of the isolated MMPI using 2D-GE IPG pH 3 to 6, 7 cm long, followed by SDS-PAGE 17.5% (*w*/*v*) acrylamide. III: Mass determination: (**E**) Size exclusion chromatography of the isolated MMPI (fraction 2 in A) performed under non-denaturing conditions, showing its mass. (**F**) Intact protein analysis of the isolated MMPI by MALDI TOF MS.

The SEC fraction 4 (Figure 1A) was further studied. Figure 2 shows the isolation and mass characterization of the *L. albus* MMPI comprising three steps: I—Isolation through high performance liquid chromatography (HPLC); II—1D and 2D electrophoretic separation of the selected peak in reducing and non-reducing conditions; and III—mass determination using SEC and MALDI-TOF MS.

Separation of the SEC fraction 4 (Figure 1) by RP-HPLC chromatography yielded different peaks, with different effects on MMP-9, with peak 2 presenting the highest inhibitory activity, as demonstrated by Figure 2B (Figure 2—I). This peak (RP-HPLC fraction 2) was further analyzed by 1D electrophoresis, under reducing and non-reducing to determine the potential presence of disulphide bonds and 2D electrophoresis. The results are shown in Figure 2C,D (Figure 2—II) and show the presence of different polypeptide bands in both conditions, suggesting an oligomer with the presence of disulphide bonds—possibly one or more interchain disulphide bonds—as judged by the difference in polypeptide patterns between lanes NR and R in Figure 2C, where the MMPI band does not present marked differences in mass when exposed to reducing or non-reducing conditions. It is noteworthy to observe that the protein band mass presented here is around 20 kDa, which seems to differ from Figure 1B which suggests a protein band of around 14 kDa; however, tricine-SDS PAGE would present a higher resolution of the low molecular weight bands, which can explain the apparent difference in masses.

For mass determination, MALDI-TOF analysis was also performed, and SEC chromatography was also used in order to identify its molecular mass (Figure 2—III). The MALDI-TOF analysis of the selected MMPI fraction is shown in Figure 2F and shows the presence of two groups of approximately 13 and 17 kDa mass fragments composed of several homologous fragments with slightly different masses, which agrees with 2D analysis (Figure 2D). On the other hand, SEC mass determination showed two major peaks with a mass around 20 and 60 kDa, which could suggest the presence of an oligomer. These features make this MMPI different from the previous protein inhibitors found in legume seeds.

**Figure 3:**
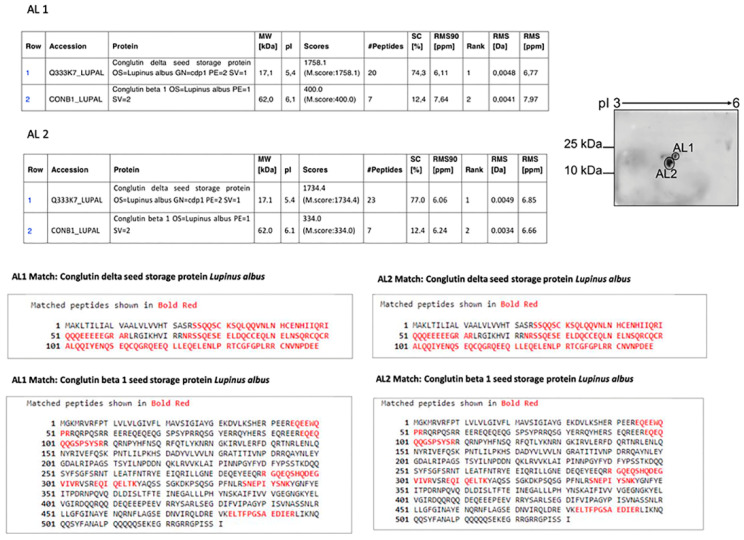
Amino-acid sequence of the *L. albus* MMPI. Mass spectrometry analysis of the isolated MMPI spots obtained from 2D analysis, AL1, and AL2, respectively, as demonstrated on the top right. The matched peptides are shown in red.

The two spots of the MMPI fraction obtained by 2D electrophoresis were named AL1 and AL2, and they were collected and sequenced by MS analysis. The sequence results for both spots are described below in Figure 3.

MS analysis subsequently demonstrated that both spots obtained from the isolated MMPI fraction (AL1 and AL2) of *L. albus* had similar compositions and comprised the same mixture of a β-conglutin fragment and δ-conglutin large chain in both spots. Since under RP-HPLC separation conditions, both β-conglutin and δ-conglutin could be denatured, and the presence of fragments of these two storage proteins is not surprising. Moreover, if we consider the microheterogeneity that characterizes legume seed storage proteins and, in particular, the extremely complex post-translation modifications that β-conglutin undergoes from it(s) precursor(s), the fragment of β-conglutin fragment is also unsurprising [22]. The fact that both groups of fragments seem to be indissociable by chromatographic and electrophoretic procedures can suggest the presence of an oligomer, although, as far as it has been described by the literature, β-conglutins and δ-conglutins are two different proteins that are evolutionary distant. Nonetheless, it is important to consider that the first initial protein fraction by SEC was not under denaturing conditions and had the predicted molecular mass whilst presenting MMPI activity, and the same was found through reverse zymography in Lima et al. [17].

On one hand, if we consider these storage protein’s features, the most likely hypothesis is that the *Lupinus* MMPI is δ-conglutin, because (a) it is the protein present in larger amounts in the MMPI fraction and (b) it holds several features that would render it a good MMPI. Belonging to the 2S sulphur-rich albumin family [23], δ-conglutin is a monomeric protein that comprises two small polypeptide chains linked by two interchain disulfide bonds: a smaller polypeptide chain, which consists of 37 amino acid residues resulting in a molecular mass of 4.4 kDa, and a larger polypeptide chain containing 75 amino acid residues with a molecular mass of 8.8 kDa [24]. The larger polypeptide chain contains two intrachain disulfide bridges and one free sulfhydryl group [24]. This could tentatively explain the slight difference in apparent molecular mass detected between R-PAGE and NR-SDS-PAGE of the isolated MMPI (Figure 2C). This protein presents specific inherent unique features among the proteins from *L. albus*: In addition to its high cysteine content, it exhibits low absorbance at 280 nm. As far as the physiological role of δ-conglutin is concerned, a storage function has been proposed for this class of proteins. However, structural similarity with the plant cereal inhibitor family, which includes bi-functional trypsin/alpha-amylase inhibitors, may suggest a defense function for this protein in addition to its storage role [25] and might corroborate its role as MMPI. Furthermore, the presence of free sulfhydryl groups in δ-conglutin could be related to a high degree of affinity towards the Zn^2+^-containing active site in MMPs and could explain its mode of inhibition. Indeed, one way to isolate δ-conglutins is by Zn precipitation [25]. Since MMP enzymes are zinc-dependent proteases, it is likely that the *L. albus* MMPI mechanism of action involves the MMP’s zinc core. This could, therefore, suggest that 2s-albumins present in the seeds of several other species, such as legumes, could have the same ability to reduce MMPs. This hypothesis is substantiated by the previous results obtained in Lima et al. [17] where similar MMPI activities, in similar molecular weight albumin fractions, were found in other legume species.

On the other hand, some features of β-conglutin might also substantiate the hypothesis that it also could play a role in MMP-9 binding. Albeit being a trimeric protein devoid of disulphide bridges in which the monomers consist of a very large number of polypeptides, glycosylated or not, ranging from 16 to over 70 kDa, a large number of post-translational proteolytic processing sites give rise to the abundance of 7S mature polypeptides observed [26]. According to Duranti, Cucchetti, and Cerletti [27], the full post-germination degradation of this protein strongly supports a storage function for β-conglutin. Nonetheless, such an extensive range of post-translation modifications is known to generate small peptides that do not end up in the composition of mature β-conglutin but have functional activities in the seed. For example, BCO (Blad-containing oligomer) is one oligomer of β-conglutin polypeptide fragments known for its potent bioactivities against fungi. It is a stable breakdown product of β-conglutin proteolysis following germination and accumulates in *Lupinus* cotyledons from days four until 12 to 14 following the onset of germination. Its major and bioactive polypeptide, Blad, is an abundant, transient polypeptide chain of 20.4 kDa, almost exactly coincident with the first cupin domain of β-conglutin precursor and displaying lectin-like activity [28]. Being highly reactive and containing two bioactive cupin domains, it is possible that there are many fragments of β-conglutin precursors (e.g., fragments of its second cupin domain) with specific uncharted activities yet to be discovered. Previous investigations in our study revealed, however, that the MMPI from *L. albus* has, unlike Blad, neither constitute antifungal nor bactericide activities (results not shown), and the sequence of the β-conglutin fragments comprising MMPI does not match that of Blad.

Another interesting fact to consider is that α- onglutins, β-conglutins and γ-conglutins (but not δ-conglutin) have been demonstrated to precipitate with Ca^2+^ and Mg^2+^ in an electrostatically dependent manner [29]. Indeed, our previous studies showed that the MMPI isolated from *L. albus* fragments can be separated with the addition of Ca^2+^ and Mg^2+^ and upon this separation both fractions lose their ability to inhibit MMP-9, but when combined once again, they regain their full MMPI inhibitory potential. This suggests that both fragments (β-conglutins and δ-conglutin) may have the potential for MMP-9 inhibition and might be more active when combined in the same fraction.

Whether a novel oligomer or not, this is still, as far as we are concerned, the first report of a direct MMP-9 inhibitory activity from legume storage proteins, rendering them different from the previous protein protease inhibitors such as serine protease Bowman–Birk inhibitors and Kunitz-type inhibitors [30] and opening up novel perspectives on the role of legumes in cancer and inflammatory diseases’ prevention.

In order to evaluate if this MMPI fraction from *L. albus* is suitable for preventive/curative diets, we tested it against MMP-2 and MMP-9 using in vitro and in vivo models.

### 2.3. The L. albus MMPI Inhibits Both MMP-2 and MMP-9 In Vitro, in a Dose-Dependent Manner, without Impairing Cell Viability or Gene Expression

**Figure 4:**
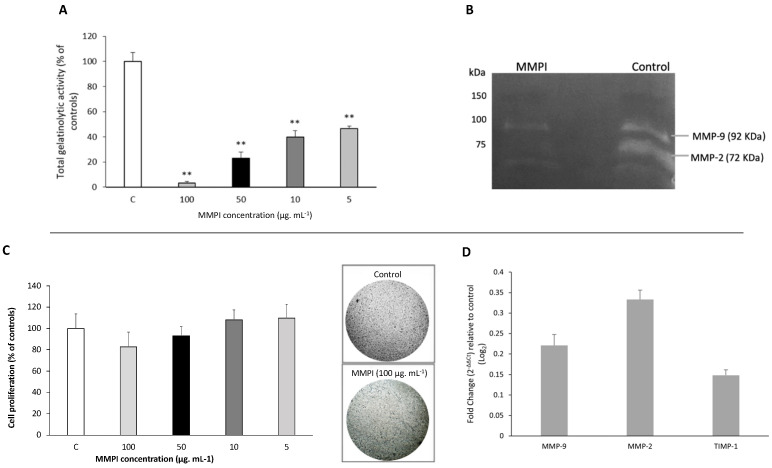
*L. albus* MMPI effect on MMP-9 and MMP-2 activity, cell proliferation, and gene expression in HT29 cells. (**A**) Dose-effect of the isolated *L. albus* MMPI on total gelatinolytic activity. The isolated MMPI was added at concentrations of 100, 50, 10, and 5 µg·mL^−1^ and gelatinolytic activity was measured by the DQ fluorogenic assay. Gelatinase activities are expressed as percentage of controls. (**B**) Representative image of the zymographic profiles of specific MMP-9 and MMP-2 activities. White bands are consistent with higher gelatinolytic activities. HT29 cells were exposed to 50 µg·mL^−1^ of the MMPI, and extracellular extracts were loaded on 12.5% (*w*/*v* acrylamide) polyacrylamide gels co-polymerized with 1% (*w*/*v*) gelatin. (**C**) HT29 cell growth after 48-hour exposure to different concentrations of the *L. albus* MMPI and representative picture of HT29 cells morphology in controls and in the highest MMPI concentration. Cells were grown in the presence of 100, 50, 10, and 5 µg protein mL^−1^ and stained with MTT. Values are expressed as a percentage of the control. (**D**) *L. albus* MMPI influence on MMP-2, MMP-9, and TIMP-1 gene expression. Cells were grown in the presence of 50 µg protein·mL^−1^ and transcripts were quantified by real-time PCR (RT-qPCR). Relative gene expression values are presented as log^2^-fold-change values in relation with the control conditions, using as reference gene Beta-actin. Data were normalized in relation to controls and the graphic is expressed as 2^−ΔΔCt^ values. All values represent the averages of at least three replicate experiments (*n* = 3) ± SD in each assay. ** *p* < 0.001 when compared to controls.

Figure 4 shows the in vitro effect of the *L. albus* MMPI on HT29 cells, namely on total and specific gelatinase activity (Figure 4A,B), cell proliferation (Figure 4C), and MMP-2 and MMP-9 gene expression (Figure 4D). A set of four different concentrations of the isolated MMPI (100, 50, 10, and 5 µg·mL^−1^) was tested in vitro for total MMP-2 and MMP-9 inhibition using the DQ-gelatin method, as displayed in Figure 4A.

Figure 4A shows that the isolated *L. albus* MMPI is able to significantly reduce the activities of both MMP-2 and MMP-9 in HT29 cells, as observed in Figure 2B, which agrees with the results obtained in Lima et al. [17]. Moreover, it very significantly inhibited total gelatinase proteolytic activity (*p* < 0.001) in a dose-dependent manner, as tested by the DQ-gelatin assay (Figure 4A), with the highest concentration of 100 µg·mL^−1^ inducing a gelatinase reduction greater than 90%. IC50 values determined for gelatinolytic inhibition were 10 µg·mL^−1^, which, when compared to other food-borne MMP inhibitors, for example, lunasine in soy [21] or some well-known MMPI phenolic compounds such as curcumine [31] or trans-resveratrol [32], *L. albus* MMPI appears to be more potent.

Figure 4C illustrates HT29 cell viability in the presence of different concentrations of the MMPI isolated from *L. albus* (100, 50, 10, and 5 µg·mL^−1^) determined after MTT staining (which can only be metabolized by living cells). Results show that the isolated MMPI did not induce a significant reduction (*p* > 0.001) in cell growth and viability when compared to controls. Furthermore, there were no visible cytotoxic effects.

It is well known that many MMP-inhibitors act upon specific signaling pathways that participate in genetic targets involved in the specific cancer under study. For example, curcumin, a well-known food-borne MMP-9 inhibitor, acts by downregulating MMP-2 and MMP-9 expression [33] and NF-κB activity [34]; trans-resveratrol inhibits MMP-induced differentiation via the p38 kinase and JNK pathways in HTB94 chondrosarcoma cells [34,35]; and lunasin from soy downregulates MMP-2 and MMP-9 expression via FAK/Akt/ERK and NF-κB signalling pathways [21]. Although DQ-gelatin assays show a direct inhibition of MMP-9 by the lupin MMPI, we further set out to test whether it had any influence on the expression of MMP-2 and MMP-9 genes as well as their tissue inhibitor TIMP-1 in HT29 cells. Results are present in Figure 4D. The fold change in the target gene is normalized to β-actin (control gene) (ΔCT) and then reported to a control sample (untreaded) (ΔΔCT). Then, the fold change from the sample to untreated conditions is calculated (2^−ΔΔCt^), allowing us to know how many times the gene is more expressed than the control. Using this method, we plotted the graphic with the 2^−ΔΔCt^ values. When upregulated in relation to the control situation, the values are in the positive side of the Y-axis and when downregulated, they are in the negative part of the axis. Hence the represented results show a tendency to upregulate MMP-2 and MMP-9, although only MMP-2 was significantly more expressed. Since gelatin zymography clearly shows that both enzymes are being inhibited, this result could show a tendency to upstream increase MMP-9 and MMP-2 expression as a natural feedback to counter-regulate their lack of activity. TIMP-1 gene expression values, on the other hand, were not significantly altered when compared to controls and would not pose any physiological significance. These findings might, therefore, support the notion that the isolated *L. albus* MMPI certainly does not reduce MMP expression but acts directedly on both gelatinases (which agrees with our earlier results in Figure 1). This could be of significant importance as an MMPI that acts directly upon MMP-2 and MMP-9 and that has no significant effects on gene expression and cell viability is more desirable because it could yield fewer secondary effects, particularly in the case of gut-related cancer pathologies where it could act in situ.

### 2.4. The L. albus MMPI Inhibits MMP-2 and MMP-9 In Vivo and Reduces Colitis Injuries

In order to ascertain the effects of the isolated MMPI in vivo and considering that MMPIs are known as IBD reducing agents [5,8,9,10], we tested its effect on mice with TNBS-induced colitis by using oral administrations 3 h after colitis induction. Table 1 shows the effect of the isolated MMPI on colon length (cm) and on the extent of intestine injury (cm).

Results show, as expected, that the animals in the control and ethanol groups exhibited no macroscopical signs of colon injury and presented no mortality, whilst intracolonic injection of TNBS resulted in a very significant (*p* < 0.05) decrease in colon length and an increase in the extent of visible injury (ulcer formation). However, p.o. administration of the *L. albus* MMPI resulted in an overall reduction in colon inflammation, with a significant attenuation of colon length reduction (*p* < 0.05), a significant reduction in the extent of visible ulcer formation (*p* < 0.05), and a significant reduction in the degree and severity of diarrhea (*p* < 0.05). Hence, overall, these results do not only corroborate the anti-inflammatory activities of the MMPI in vivo but also demonstrate that it is able to maintain its biological activity throughout the digestive process.

**Figure 5:**
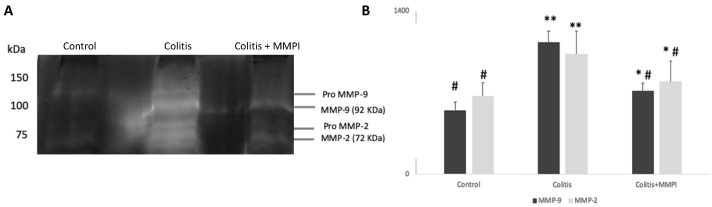
Effect of the *L. albus* MMPI administration on the colon tissue gelatinase activities of MMP-2 and MMP-9 from colitis-induced mice. (**A**): Representative image of the zymographic profiles of MMP-9 and MMP-2 activities of the colons. Protein extracts of the colon were loaded on 12.5% (*w*/*v* acrylamide) polyacrylamide gels co-polymerized with 1% (*w*/*v*) gelatin. (**B**): Densitometric analysis of the gelatinolytic activity of MMP-9 and MMP-2 obtained in the zymographies. Control group (*n* = 6); Colitis group (*n* = 10); Colitis + MMPI =colon from animals treated with *L. albus* MMPI (15 mg·kg^−1^, *n* = 9, p.o.). Results are average of at least three replicates. ^#^ *p* < 0.05 vs. Colitis; * *p* < 0.05 vs. Control and ** *p* < 0.001 vs. Control.

The activity of MMP-2 and MMP-9 in the observed colons is depicted in gelatin zymography Figure 5A and in the respective densitometric analysis (Figure 5B).

Results demonstrated that, as expected, TNBS-induced colitis significantly increased MMP-9 and MMP-2 activities (*p* < 0.05), demonstrated by the high intensity of the white bands, in both the active as well as in the inactive forms of the enzymes, whereas in controls a low activity of the active forms of MMP-2 and MMP-9 was observed (Figure 5A). With *L. albus* MMPI oral administration, there was an evident significant reduction in MMP-2 and MMP-9 activities (*p* < 0.05), but particularly in their active form, when compared to the TNBS group. These results are consistent with the data obtained from Table 1 and corroborate that oral administration of the *L. albus* MMPI inhibited the colitis-induced rise in gelatinolytic activities observed in animal models, leveling them to physiological and morphological levels closer to those observed in healthy controls. Furthermore, the fact that the isolated MMPI maintained its biological activity throughout the digestive process in oral treatments substantiates its potential use in a dietary approach.

### 2.5. L. albus MMPI May Present the Features of an Ideal MMP-9 Inhibitor for Gut-Related Diseases

The discovery that gelatinases are highly upregulated in several pathological processes has spurred the development of MMP inhibitors, whilst rendering them attractive targets for therapeutic intervention. However, a long history of unsuccessful clinical trials has demonstrated limited clinical utility for MMPIs because of several setbacks: generalized size-effects, dose-limiting toxicity, and severe side effects. One method to surpass these setbacks would be to use MMPIs capable of acting directly in loco, particularly in gut-related pathologies where the ingestion of specific MMP-2 and MMP-9 inhibitors could act directly within the digestive tract without producing any side effects. However, some major limitations should still be surpassed, such as chemical destruction during the digestive process and absorption to the blood flow, thus inducing generalized effects. The findings presented in this study seem to suggest that novel *Lupinus* MMPI may hold the features of an ideal MMPI for dietary approaches against gut-related ailments, naturally excluding people who suffer from allergy to lupin’s conglutins.

Previous results show that the fragments produced by this method are highly soluble in water and resistant to boiling heat, making it feasible to isolate them in larger quantities [19].

Our present results show that *L. albus* MMPI resists digestion and it is effective in low amounts while presenting no toxicity (hence, being suitable to use in dietary approaches) and it apparently inhibits gelatinases directly; hence, it is a good candidate for in situ approaches, particularly in the case of gastrointestinal pathologies related to aberrant gelatinase activities, such as inflammatory bowel diseases, as has been described for previous MMP inhibitors [3,8]. Although much work must be performed still, these features may prove further useful as a nutraceutical or in functional foods in the prevention and treatment of a very wide array of diseases related to MMP-9 activity.

## 3. Materials and Methods

### 3.1. Protein Isolation and Identification

Dry, mature seeds of *Lupinus albus* L. (lupin) were used in this study. The total soluble protein from approximately 100 g ± 0.1 g of dry lupin cotyledons (i.e., seed without embryo and tegument) was extracted by using 50 mM of Tris-HCl buffer, pH 7.5 (1:10, *w*/*v*). The homogenate was centrifuged at 13,500× *g* for 30 min at 4 °C.

### 3.2. Fast Protein Liquid Chromatography

Protein samples were fractionated by Fast Protein Liquid Chromatography (FPLC; GE Healthcare Life Sciences) size-exclusion chromatography (SEC) and injected into a Superdex TM 75 HR 10/30 column (GE Healthcare Life Sciences) and equilibrated with degassed 100 mM Tris-HCl buffer, pH 7.5, at a flow rate of 0.5 mL/min. Protein peaks eluted from the column were detected at 280 nm. All collected fractions were tested for their inhibitory activity on MMP-9 by using a DQ-gelatin assay (please see methods below).

For molecular mass estimation, the ÄKTA Pure chromatography system with the UV detector (GE Healthcare Life Sciences) was used. Gel filtration was performed on a Superose 12 HR 10/30 column (GE Healthcare Life Sciences) equilibrated with 100 mM Tris HCl buffer, pH 7.5, containing 50 mM NaCl. Proteins were eluted at 0.5 mL·min^−1^ and the protein fractions were desalted twice on NAP-10 columns (GE Healthcare Life Sciences), lyophilized, and stored at −20 °C until required. The calibration curve was prepared with blue dextran, bovine serum albumin, chymotrypsin, and cytochrome c. All chromatographic steps were conducted at room temperature.

### 3.3. High-Performance Liquid Chromatography

The MMPI fraction isolated by FPLC-SEC and identified by the DQ-gelatin assay was fractionated in a High-Performance Liquid Chromatography (HPLC) device (Waters 2695 Separations Module) equipped with a Waters 2998 Photodiode Array Detector. Protein samples were separated in a C18 reverse phase column, Zorbax 300SB 5 µm, 250 mm × 4.6 mm. The elution was made with eluent A (0.1 % *v*/*v* Trifluoroacetic Acid (TFA)) and solvent B (acetonitrile in 0.1 % *v*/*v* TFA). Peak detection was made at 214 nm and 280 nm.

### 3.4. 1-D Electrophoresis

SEC protein fractions were separated by Tricine-SDS-PAGE in 12.5 polyacrylamide gels. Electrophoresis was performed in a vertical system, the Anode buffer was Tris-HCL, 200 mM, pH 8.9, and the cathode was 100 mM Tris, 100 mM Tricine, and 1% SDS (*m*/*v*). Samples were lyophilized and resuspended in sample buffer of 1% SDS (*m*/*v*), 8 M Urea, 1% (*v*/*v*), 1% (*v*/*v*) 2-mercaptoethanol, and 0.01 M Tris (adjusted to pH 6.8 with phosphoric acid). Samples were incubated at 60 °C for 15 min, and electrophoresis was performed at 200 V and 20 mA per gel. Protein bands were detected with Comassie G Staining, following standard procedures.

The HPLC isolated MMPI (50 μg·mL^−1^) was loaded onto a 17.5% (*w*/*v* acrylamide) polyacrylamide gel with denaturing, reducing buffer (100 mM Tris-HCl buffer, pH 6.8, containing 100 mM β-mercaptoethanol, 2% *w*/*v* SDS, 15% *v*/*v* glycerol, and 0.006% *w*/*v* m-cresol purple) or denaturing, non-reducing buffer (100 mM Tris-HCl buffer, pH 6.8, containing 2% *w*/*v* SDS, 15% *v*/*v* glycerol, and 0.006% *w*/*v* m-cresol purple). One-dimensional electrophoresis was carried out following the method described by Lima et al. [17].

### 3.5. 2-D Electrophoresis

The 2D-gel electrophoresis and spot digestions were carried out as described previously [36]. Isoelectric focusing IPG-Strips pH 3 to 6, 7 cm long and 17.5% (*w*/*v*) acrylamide SDS-PAGE gels were used.

### 3.6. MALDI-TOF TOF

The intact isolated MMPI was analyzed by MALDI TOF MS using an Ultraflex II MALDI-TOF TOF Bruker-Daltonics equipped with a LIFT cell and N2 laser. The mass spectrometer was operated with positive polarity in linear mode and spectra were acquired in the range of *m*/*z* 5000–20,000. A total of 1000 spectra were acquired at each spot position at a laser frequency of 50 Hz. External calibration was performed using a protein calibration standard I from Bruker: [M + H]^+^ of insulin (5734.51 *m*/*z*), ubiquitin I (8565.76 *m*/*z*), cytochrome c (12,360.97 *m*/*z*), and myoglobin (16,952.30 *m*/*z*); [M + 2H]^2+^ of cytochrome c (6180.99 *m*/*z*) and myoglobin (8476.65 *m*/*z*).

### 3.7. MS/MS Analysis

Prior to ESI MS/MS analysis, all samples were diluted with 100 μL of 0.1% (*v*/*v*) aqueous formic acid containing 3% (*v*/*v*) acetonitrile before loading onto an EASY-nLC II equipped with an EASY-column, 2 cm, ID 100 μm, 5 μm, C18-A1 (Thermo Fisher Scientific) and an EASY-column, 10 cm, ID 75 μm, 3 μm, C18-A2 (Thermo Fisher Scientific). Chromatographic separation was carried out using a multistep linear gradient at 300 nL/min (mobile phase A: aqueous formic acid 0.1% *v*/*v*; mobile phase B: 90% *v*/*v* acetonitrile and 0.1% *v*/*v* formic acid), 0–90 min linear gradient from 0% to 35% of mobile phase B, 90–115 min linear gradient from 35% to 90% of mobile phase B, and 115–120 min isocratic flow at 90% of mobile phase B.

Selected isolated peaks were analyzed on a 5600 TripleTOF mass spectrometer (ABSciex^®^) in information-dependent acquisition (IDA) mode. Peptides were resolved by liquid chromatography (nanoLC Ultra 2D, Eksigent^®^) on a MicroLC column ChromXPTM C18CL reverse phase column (300 μm ID × 15 cm length, 3 μm particles, 120 Å pore size, Eksigent^®^) at 5 μL·min^−1^. Peptides were eluted into the mass spectrometer with a multistep gradient: 0–2 min linear gradient from 5 to 10%, 2–45 min linear gradient from 10% to 30% and, and 45–46 min to 35% of acetonitrile in 0.1% (*v*/*v*) TFA. Peptides were eluted into the mass spectrometer using an electrospray ionization source (DuoSpray™ Source, AB Sciex) with a 50 μm internal diameter stainless steel emitter (New Objective). Protein identification was obtained using Protein Pilot™ software (v 5.0, ABSciex^®^).

### 3.8. MMP-9 and MMP-2 Catalytic Activities

#### 3.8.1. DQ-Gelatin Assay

The fluorogenic substrate DQ-gelatin assay was performed as described by Lima et al. [17].

#### 3.8.2. Gelatin Zymography

In order to determine specific metalloproteinase activities, gelatin-zymography was performed according to standard methods [17,37], with the following modifications: SDS-polyacrylamide gels (12.5% *w*/*v* acrylamide) were copolymerized with 1% (*w*/*v*) gelatin. HT29 cancer cell culture supernatants treated with a non-reducing buffer containing 62.6 mM Tris-HCl pH 6.8, 2% (*w*/*v*) SDS, 10% (*v*/*v*) glycerol, and 0.01% (*w*/*v*) bromophenol blue were loaded into each well of the SDS-gel. Electrophoresis was carried out as described before [17] in a 12.5% (*w*/*v*) acrylamide resolving gel and a 5% (*w*/*v*) acrylamide stacking gel, performed in a vertical electrophoresis unit at 200 V and 20 mA per gel. After electrophoresis, gels were washed three times in 2.5% (*v*/*v*) Triton X-100 for 60 min each and were incubated 48 h with a specific buffer solution (50 mM Tris-HCl pH 7.4, 5 mM CaCl2, 1 µM ZnCl2, and 0.01% *w*/*v* sodium azide), stained with Coomassie Brilliant Blue G-250 as described by Lima et al. [17]. White bands visible against a blue background marked the gelatinase activity of each proteinase [37]. Protein band intensities were determined by densitometry, as described previously [17].

### 3.9. In Vitro Colon Cancer Cell Assays

#### 3.9.1. HT29 Cell Cultures

The human colon adenocarcinoma cell line, HT29 (ECACC 85061109), obtained from a 44-year-old Caucasian female was used. HT29 cells were maintained according to Lima et al. [17].

#### 3.9.2. Cell Viability Assay

HT29 cultured cells were seeded in 96-well plates (2 × 10^4^ cells/well) and MMPI sample was added to the growth medium at different concentrations (100, 50, 10, and 5 μg·mL^−1^) concentration and incubated for 48 h. The extracellular medium was collected, and the wells washed with PBS to remove unattached cells. Cell proliferation and viability was determined using the standard MTT assay as described before, with few alterations [17]. Briefly, 50 µL of serum-free media and 50 µL of MTT solution were added into each well and incubated at 37 °C for 3 h. After incubation, 100 µL of DMSO was added into each well. Absorbance was read at 590 nm.

### 3.10. Assessment of Gene Expression by Quantitative Real-Time PCR (RT-qPCR)

#### RN Extraction and cDNA Synthesis

Total RNA was extracted from HT29 cells using the NZY Total RNA Isolation kit as described by the manufacturer. Quantification was carried out in a Synergy HT Multiplate Reader, with Gene5 software, using a Take3™ Multi-Volume Plate (Bio-Tek Instruments Inc., Winooski, VT, USA). For reverse transcription, the RevertAid reverse transcriptase priming with oligo-d(T) kit was used according to the manufacturer’s recommendations (Thermo Scientific, Waltham, MA, USA).

For each gene studied (MMP-9, MMP-2, and TIMP1), a set of specific primers was designed and used to amplify HT29 cDNA resulting from the transcription of 2 µg of total RNA by using conventional PCR and gel agarose electrophoresis. When amplification was observed, confirming the expression, the transcripts were quantified by real-time PCR (RT-qPCR), which was performed in 20 μL reaction volumes composed cDNA derived from 2 µg RNA and 0.5 μM gene-specific primers (Online Resource 1) in SsoFast™ EvaGreen^®^ Supermixes (Bio-Rad, Hercules, CA, USA) using an iQ5 Real-Time Thermal Cycler (BioRad, Hercules, CA, USA). Reaction conditions for cycling were as follows: 95 °C for 3 min followed by 40 cycles at 95 °C for 10 s, 61 °C for 25 s, and 72 °C for 30 s. Each analysis was performed in triplicate reactions of three biological replicates. The corresponding quantification cycles (Cq) were determined by the iQ5 optical system software (Bio-Rad, Hercules, CA, USA) and exported to a MS Excel spreadsheet (Microsoft Inc., Redmond, WA, USA) for further analysis. Cq values of each gene of interest were normalized with respect to actin (Act) [38].

### 3.11. In Vivo Animal Model of Colitis

#### 3.11.1. Animals

Male CD-1 mice, 25 to 30 g in weight and 5 to 6 weeks of age (Harlan Iberica, Barcelona, Spain), were housed in standard polypropylene cages with ad libitum access to food and water inside a controlled environment room kept at 22 °C ± 1 °C with a 12 h light and 12 h dark cycle at the Faculty of Pharmacy Central Animal Facility, University of Lisbon, Lisbon, Portugal.

#### 3.11.2. Animal Care and Maintenance

Experiments were performed by agreeing to the most recent rules and recommendations for the care and processing of laboratory animals, namely to the presently adopted European Commission regulations (Directive 2010/63/EU). In addition, the studies were performed in agreement with the ARRIVE Guidelines for Reporting Animal Research. The Ethics Committee of the Faculty of Pharmacy (University of Lisbon) also endorsed the experimental protocol (0019/2018; date of approval: 27 February 2018).

#### 3.11.3. Induction of Colitis

TNBS was instilled as an intracolonic single dose as previously described [39]. On induction day (day 0), mice were anesthetized with ketamine (100 mg·kg^−1^) and xilazine (10 mg·kg^−1^). Then, 100 µL of TNBS solution was administered with a catheter inserted 4.5 cm into the colon. Mice were kept for 30 min in a Trendelenburg position to avoid reflux. Four days after induction, mice were euthanized by cervical dislocation and necropsied. The colon was removed, freed from surrounding tissues, and opened longitudinally for observation and classification of diarrhea severity. Subsequently, the colon was washed with PBS for macroscopical observation of the tissue.

#### 3.11.4. Experimental Groups

Animals were randomly allocated into four experimental groups as described below.

1. Control group (*n* = 6): Animals were subjected to the procedures described above except for intracolonic administration which comprised 100 µL of saline solution. During the 4 days of the protocol, the animals were administered orally with 10 mL·kg^−1^ of distilled water.

2. Ethanol (EtOH) group (*n* = 6): Animals were subjected to the procedures described above except for intracolonic administration with 100 μL of 50% (*v*/*v*) ethanol. During the 4 days of the protocol, the animals were administered orally with 10 mL·kg^−1^ of distilled water.

3. TNBS group (*n* = 10): Animals were administered with 100 µL of 2.5% (*w*/*v*) TNBS in 50% (*v*/*v*) ethanol. During the 4 days of the protocol, the animals were administered orally with 10 mL.kg^−1^ of distilled water.

4. TNBS + Lupinus MMPI p.o. (*n* = 9): Animals were administered with 100 µL of 2.5% (*w*/*v*) TNBS in 50% (*v*/*v*) ethanol. During the 4 days of the protocol, the animals were administered orally with the isolated MMPI (15 mg·kg^−1^).

Oral administrations were performed daily by gastric gavage, starting from 4 h after the initial administration of TNBS.

#### 3.11.5. Macroscopic Evaluation of Colitis Severity

After colon removal, a longitudinal incision was performed for observation of content and classification of diarrhea severity by an observer blinded regarding the experimental groups. Afterwards, the colon was rinsed with saline and analyzed with a surgical microscope for closer observation of the tissues. The colon length was then measured, as well as the extent of the injuries (if present).

#### 3.11.6. Gelatin Zymography of Colon Extracts

Extraction of colon proteins was performed according to Castaneda et al. [40]. In order to determine specific metalloproteinase activities in colon protein supernatants, a gelatin-zymography was performed as described before [17] with the following modifications: SDS-polyacrylamide gels (12.5% *w*/*v* acrylamide) were copolymerized with 1% (*w*/*v*) gelatin. Colon polypeptide extracts previously treated with a denaturing, non-reducing buffer containing 62.6 mM Tris-HCl pH 6.8, 2% (*w*/*v*) SDS, 10% (*v*/*v*) glycerol, and 0.01% (*w*/*v*) bromophenol blue were loaded into each well of the SDS-gel. Electrophoresis was carried out as described before (Section 3.8.2).

### 3.12. Statistical Analysis

All experiments were performed in triplicate in at least three independent times and the data are expressed as the mean ± standard deviation (SD). SigmaPlot software (version 12.5) was used for comparing different treatments, using one-way and two-way analysis of variance (ANOVA). Tukey’s test was used to compare differences among groups and the statistical differences with *p* values lower than 0.05 were considered statistically significant.

## 4. Conclusions

Here, we have shown novel MMP-2 and MMP-9 inhibitory activity from protein components of lupine, which were found to be active in vivo when administered orally, whilst alleviating the symptoms of induced colitis in mice. These results open novel perspectives on the role of storage proteins in *Lupinus* and other legume seeds as possible nutraceutical or functional foods in the prevention and treatment of diseases related to MMP-9 activity.

## Figures and Tables

**Table 1:** Morphological and functional observations in clean and transversely opened colons after harvest. Effect of the *L. albus* MMPI administration on the length of colon (cm). Sham group (*n* = 6), TNBS group (*n* = 10); TNBS + MMPI p.o. (15 mg·kg^−1^; *n* = 9). The severity scoring of diarrhea is as follows: normal = 0; lightly soft stools = 1; soft stools = 2; liquid stools = 3. ^#^ *p* < 0.05 vs. Control; * *p* < 0.05 vs. TNBS.

	Length of Colons	Size of Lesion	Presence/Consistency of Diarrhea
Control	14.5 ± 0.082	0	0
TNBS + EtOH 50%	11.8 ± 0.19 ^#^	3.6 ± 0.14 ^#^	3 ^#^
TNBS + MMPI p.o.	14.8 ± 0.33 *	2.44 ± 0.84	1.13 ± 0.35 *

## Data Availability

All data are available in the manuscript.

## Patent

DEFLAMIN: Therapeutic protein. PCT International Patent Application No. PCT/EP2017/075020. Filed on 30 September 2017. Available at https://patentscope.wipo.int/search/en/detail.jsf?docId=WO2018060528&recNum=4&office=&queryString=FP%3A%28075020%29&prevFilter=&sortOption=Pub+Date+Desc&maxRec=48 (accessed on 20 May 2021).
